# 252. Alveolar Macrophages Modulate Host Inflammatory Responses to Pulmonary Melioidosis

**DOI:** 10.1093/ofid/ofad500.324

**Published:** 2023-11-27

**Authors:** Sarah M Baker, Timothy E West

**Affiliations:** University of Washington, Seattle, Washington; University of Washington, Seattle, Washington

## Abstract

**Background:**

Melioidosis is an emerging infection caused by the facultatively intracellular pathogen and Tier 1 select agent *Burkholderia pseudomallei (Bps).* Pneumonia, the most common manifestation of melioidosis, predicts poor prognosis, yet the pulmonary immune response to *Bps* is poorly understood. Alveolar macrophages (AMs) are innate immune cells in the lung and are distinct from monocytes and other macrophages in their response to pathogens, yet their role in host defense against melioidosis is unknown. We sought to determine if AMs modulate bacterial replication, dissemination, pulmonary inflammatory response, and health outcomes to *Burkholderia* using a murine model of melioidosis.

**Methods:**

To deplete mice of AMs, we instilled 100μl clodronate-liposomes or PBS-liposomes intratracheally into C57Bl/6 mice and confirmed AM depletion by flow cytometry. Twenty-four hours after AM depletion, we infected mice by aerosolization with a sub-lethal dose of 7.8 x 10^3^ CFU/lung of *B. thailandensis* (*Bt*), a BSL-2-compliant surrogate model of *Bps* infection. At 24 and 48 hours after infection we quantified pulmonary bacterial burdens, bacterial dissemination to the spleen, lung homogenate cytokines, and clinical markers of illness (health score, weight, and temperature) in mice depleted of AMs and compared these to sham-depleted mice.

**Results:**

Mice deplete of AMs had fewer bacteria in the lungs 24 hours after infection and similar numbers of bacteria at 48 hours; there were no differences in bacterial dissemination or clinical health outcomes. Mice deplete of AMs produced less of the inflammasome-associated cytokines IL-18 and IL-1β at 24 and 48 hours and produced less of the pro-inflammatory cytokine TNF-α

and the neutrophil chemokine MIP-2 at 24 hours. When adjusting for lung bacterial CFUs, mice deplete of AMs still produced less IL-1β at 24 hours.

**Conclusion:**

In a murine model of sub-lethal pulmonary melioidosis, AMs do not contribute to early bacterial restriction and may instead act as an intracellular *Burkholderia* replication niche or otherwise facilitate replication in the lung. However, AMs are likely to be a main source of IL-1β in the lung, signifying their relevance in the canonical inflammasome pathways implicated in host response melioidosis.

**Disclosures:**

**All Authors**: No reported disclosures

